# Medical Cannabis Utilization Across States With Varying Legal Status

**DOI:** 10.1016/j.focus.2025.100396

**Published:** 2025-07-22

**Authors:** Deanna Romero, Thomas A. Clobes, Marshall M. Mee, Isaac Quintanilla Salinas

**Affiliations:** 1 Department of Health Sciences, California State University Channel Islands, Camarillo, California; 2 Department of Psychology, California State University Channel Islands, Camarillo, California; 3 Department of Mathematics, California State University Channel Islands, Camarillo, California

**Keywords:** Medical cannabis, health policy, cannabis legalization, medical cannabis access

## Abstract

**Introduction:**

Despite increasing legalization of medical cannabis across the U.S., significant barriers to access and utilization remain, particularly in states with restrictive policies. This study aimed to explore the patterns of medical cannabis use, methods of acquisition, perceived efficacy, and barriers to use, with a focus on how these factors vary by state-level legal status.

**Methods:**

A cross-sectional, nationwide survey was administered online during August–September 2023 to a purposefully sampled U.S. adult population. Of 2,824 respondents, 996 individuals self-identified as using cannabis for medical purposes and were included in the final analysis. Measures included demographic variables, cannabis usage patterns, therapeutic indications, perceived effectiveness, and challenges related to legal frameworks and healthcare disclosure.

**Results:**

Cannabis use varied significantly by state legal status (chi-square=22.26, *p*=0.001), with 43.1% of respondents in fully legal states using medical cannabis, compared with 27% in prohibition states. Anxiety (65.9%), depression (48%), and chronic pain (37%) were the most commonly reported conditions. Participants reported diverse consumption methods, with smoking (74.3%) being the most common, although also associated with the highest rate of side effects (chi-square=216, *p*<0.001). Overall, 32.3% of participants rated medical cannabis as a great deal effective for their primary condition. Legal status also influenced the conditions treated: bipolar disorder was more frequently reported in prohibition states, whereas degenerative neurologic conditions were more common in legal states. Notably, 43.2% of medical cannabis users had not disclosed their use to a primary care provider. Of those, nearly 40% cited fear of provider disapproval, and 27.1% cited concerns about legality—even in states where medical cannabis was permitted. In addition, 45% of those who experienced barriers cited cost as a deterrent, and over half reported using less medical cannabis owing to these challenges.

**Conclusions:**

The findings highlight significant variations in medical cannabis usage and associated challenges based on state legal frameworks. Barriers such as cost and fear of disclosure underscore the need for clearer communication and policy reforms to improve access and patient–provider dialog. Further research is warranted to explore the clinical implications of medical cannabis use across diverse populations.

## HIGHLIGHTS

• Medical cannabis use varies significantly by state-level legalization status.
• Legal concerns deter patients from disclosing cannabis use to healthcare providers.
• Smoking remains the dominant method despite higher side-effect prevalence.
• Anxiety, depression, and insomnia are the most common medical cannabis indications.
• State legality influences the conditions treated with medical cannabis, such as bipolar disorder.

## INTRODUCTION

Since the passage of the Controlled Substance Act in 1970, cannabis has been classified as a Schedule I controlled substance by the Drug Enforcement Administration in the U.S.—formally indicating it as having a high potential for abuse and misuse and no accepted medical use.^1^ However, in May 2024, the HHS proposed a reclassification of cannabis from Schedule I to Schedule III after their determination that cannabis does possess accepted medical indications.^2^ In addition, there is a notable shift in healthcare professionals’ perspectives toward the medicinal use of cannabis for various conditions.^3^ Consequently, ongoing research is focused on further investigating the implications of cannabis use in light of these evolving perceptions.

As of early 2025, 11 U.S. states maintain full prohibition of cannabis other than products containing low levels of delta-9-tetrahydrocannabinol.^4^ Despite limited access in states where cannabis remains illegal, cannabis use has escalated since the commercialization of cannabis for nonmedical use in the U.S.^5^ Given the varying legal status of cannabis throughout the U.S., the potential for variation in usage patterns and other factors in different states exists.

Evidence suggests that cannabis may offer a range of therapeutic benefits across various clinical domains.^6^ Historically utilized for centuries, cannabis has recently undergone more rigorous scientific investigation to elucidate both its observable and perceived therapeutic effects.^7^ Although not typically a first-line treatment, cannabis presents a valuable clinical alternative for managing acute and chronic conditions, particularly when conventional therapies prove insufficient. Although the risks associated with cannabis use have been documented, data indicate that these risks may have diminished as cannabis use has become more widespread.^8^ The proposed rescheduling of cannabis to a less restrictive drug class is anticipated to facilitate increased clinical research, thereby enhancing the understanding of its pharmacological properties and potential as a primary therapeutic option in clinical practice.

The prohibition of cannabis in states where it remains fully illegal limits access for individuals who might derive benefits from its use. In the relatively unexplored domain of cannabis medicine, numerous patients have reported symptom relief for both chronic and acute conditions.^9^ These conditions can vary widely in severity, from mild to debilitating.^9^ Furthermore, research has suggested that cannabis could serve as a viable alternative to opioids, particularly in contexts characterized by opioid misuse and abuse.^10^

Notably, significant data exist on the use of cannabis for nonmedical reasons.^11^, ^12^, ^13^ Cannabis use has increased, and concentrations of delta-9-tetrahydrocannabinol have increased significantly over the past several decades.^14^^,^^15^ Despite the literature available on cannabis usage patterns, there is a lack of an understanding of how state laws impact medical cannabis (MC) use specifically.

Considering the potential medical applications of cannabis and varying legal landscape throughout the U.S., the objective of this research study was to understand MC use and the impact of cannabis policies. This survey was designed to gather data regarding acquisition methods, frequency of use, and indications for use across the U.S. The data were considered in relation to the state-level policies regarding MC use.

This survey examined several areas regarding participants’ use of MC. Respondents provided demographic information, MC usage patterns, side effects experienced, and other information on their MC use. Examining the impact of legalization involves assessing various factors such as changes in MC prevalence, consumption, and usage trends.

## METHODS

### Study Sample

The national survey, taken anonymously, was conducted using the InnovateMR platform, a reputable company specializing in survey sampling. InnovateMR has over 1 million panelists, allowing for a diverse, representative sample, and has been used extensively in previous survey-based research.^16^, ^17^, ^18^ A purposeful sampling technique was used to garner data from a sample representative of the U.S. in terms of race and legal access to MC. The survey was available online during August and September 2023. In total, 22,373 respondents aged ≥18 years were invited through the InnovateMR platform and/or email to participate in the survey, which took approximately 20 minutes to complete. Participation was voluntary; participants received compensation consistent with their specific agreement with InnovateMR. To be eligible for the survey, respondents had to be aged ≥18 years and live within the U.S.; to make comparisons of disclosed cannabis use between participants who lived in states with various legal status, initial analyses included all who completed the survey. To be eligible for the final analysis, respondents had to self-identify as using cannabis for medical purposes, regardless of whether it was with a physician recommendation.

There were 2,844 participants who responded to the survey, with 1,106 (38.9%) reporting not using cannabis ever and 1,738 (61.1%) reporting using cannabis either currently or in the past for either medical or nonmedical use; however, 20 individuals did not reside in the U.S. and were removed from analysis. Of those using cannabis, 781 (44.9%) reported using cannabis for nonmedical use only, 277 (15.9%) reported using cannabis for medical purposes only, and 680 (39.1%) reported using cannabis for both medical and nonmedical use. Of those who reported not using any cannabis, 460 (41.6%) participants previously used cannabis for nonmedical use, 37 (3.3%) participants previously used cannabis for medical use, and 72 (6.5%) previously used cannabis for medical and nonmedical use. Therefore, 1,066 who commenced the survey were eligible, but 70 participants either did not complete the survey or failed an attention check question. Thus, the final study sample contained 996 (34.5%) participants who used cannabis for medical purposes, passed all the attention checks throughout the survey, and lived in the U.S.

In the full sample (N=2,824) of MC users, there were 2,055 (72.8%) participants who identified as White, 572 (20.3%) who identified as Black/African American, 110 (3.9%) who identified as American Indian/Alaskan Native, and 99 (3.5%) who identified as Asian. There were 385 (13.6%) participants who identified as of Hispanic origin. A total of 1,009 (35.7%) participants identified as male; 1,791 (63.4%) identified as female; and 21 (0.7%) identified as nonbinary/third gender. There were 2,423 (85.8%) participants who identified as heterosexual, with the remaining 351 (12.4%) identifying as gay, lesbian, or bisexual. There were 999 (35.3%) participants who were single/never married, 336 (11.9%) in a domestic partnership, 974 (34.5%) married, 411 (14.5%) separated/divorced, and 104 (3.7%) widowed. A total of 2,683 (95%) participants were U.S. citizens by birth.

In the final sample of MC users, there were 778 (78.1%) participants who identified as White, 162 (16.3%) who identified as Black/African American, 52 (5.2%) who identified as American Indian/Alaskan Native, and 24 (2.4%) who identified as Asian. There were 144 (14.5%) participants who identified as of Hispanic origin. A total of 343 (34.3%) participants identified as male, 642 (64.5%) identified as female, and 10 (1%) identified as nonbinary/third gender. There were 835 (83.8%) participants who identified as heterosexual, with the remaining 161 (16.2%) identifying as gay, lesbian, or bisexual. There were 320 (32.1%) participants who were single/never married, 146 (14.7%) in a domestic partnership, 331 (33.2%) married, 160 (16.1%) separated/divorced, and 39 (3.9%) widowed. A total of 967 (96.1%) participants were U.S. citizens by birth.

### Measures

This study employed a cross-sectional design to explore the prevalent medical conditions for which adult patients with MC use in the U.S. who utilize cannabis as a therapeutic intervention as well as measures of quantities utilized, routes of administration, and perceived side effects from the medication. The survey questions were designed with clinician and MC professional input. Respondents self-identified as patients on MC, defined as “cannabis used to treat or aid with some sort of medical issue, whether or not with a recommendation or supervision from a medical provider.” Participants were then asked a series of questions regarding their experiences with MC—the full list of questions can be reviewed in the Appendix (available online). Participants were asked to identify the primary ailment that they used MC for and many questions related to the identified ailment. For participants who indicated that more than 1 ailment was what they primarily used their cannabis for, the survey automatically assigned the ailment questions to the ailment the least number of respondents had previously indicated. Either way, the questions clearly indicated which ailment was being asked about.

Throughout the survey, there were attention check questions ensuring that respondents were reading the questions thoroughly and were fully engaged. Participants received 2 or 3 attention check questions. If participants failed any of the attention check questions, they were automatically withdrawn from the survey.

### Statistical Analysis

Participants were divided into 3 segments of cannabis legalization on the basis of state of residence: (1) medically and nonmedically legal, (2) only medical-use legal, and (3) neither legal.^4^ Counts and proportions were reported for survey questions with categorical responses. The mean with SD was reported for survey questions with numerical responses. Several questions in the survey were analyzed to see whether there is a significant association with the state’s legal status for cannabis. A chi-square independence test was used to test the association for questions with categorical responses, and a Kruskal–Wallis test was used for questions with numerical responses. A Monte-Carlo Fisher’s exact test, a simulation-based hypothesis test alternative to standard chi-square tests when the assumptions are not met, was used in questions with small samples in categories. All analysis was conducted in R 4.4.1.^19^

Participants electronically acknowledged an informed consent prior to beginning the survey. The study was reviewed and approved by the California State University Channel Islands IRB.

## RESULTS

Although this analysis focuses on 996 MC users, to determine usage variation between states with different legal status, the 2,824 participants who began the survey and reside in the U.S. were compared. There is a significant association between cannabis (nonmedical, medical, nonmedical and medical, and none) use and state legal status (chi-square=22.26, *p*=0.001). Table 1 provides the cross-tabulation between state legal status and cannabis use. In addition, there is a significant association between MC use (versus none or nonmedical use) and state legal status (chi-square=7.47, *p*=0.023). States with legal access to MC and non-MC had 457 (43.1%) participants who use MC, whereas states that prohibit cannabis use completely had 287 (27%) participants who use MC.

**Table 1:** The Cross-Tabulation Between State Legal Status and Cannabis Use

Cannabis use	Medical/recreational use allowed	Medical use only	No access	Total
None	198 (37.2%)	170 (32.0%)	164 (30.8%)	532 (100%)
Yes, medical use only	126 (41.5%)	108 (34.7%)	77 (24.8%)	311 (100%)
Yes, recreational and medical	331 (44,1%)	209 (27.9%)	210 (28.0%)	750 (100%)
Yes, recreational use only	526 (42.7%)	307 (24.9%)	398 (32.3%)	1,231 (100%)
Total	1,181 (41.8%)	794 (28.1%)	849 (30.1%)	2,824 (100%)

*Note:* Cannabis use is classified as medical use only, recreational or medical use, and recreational use only. Row proportions are utilized. The table demonstrates the type of cannabis use of all participants of the survey, by state legality status. Respondents who answered *Yes* to medical use only, recreational and medical use, and recreational use only were used for further analysis in the study. The group frequency and proportion (inside the parentheses) were reported. The proportion represents the number of participants that stated *Yes* versus *No* in the row and column grouping.

Several of the variables were tested to see whether the legality of cannabis use in the state of residence for the participant had an effect on the responses. For context, 427 (42.9%) participants lived in a state that allowed medical and nonmedical (adult) use, 296 (29.7%) participants lived in a state where only MC is allowed, and 273 (27.4%) participants lived in a state with no legal access. The majority of survey questions did not have a significant association with state cannabis legality status at a significance level of 0.05, as detailed below.

When considering challenges in obtaining MC, the plurality of participants, 467 (46.9%), reported not having any problems obtaining it. Of those who experienced challenges in obtaining MC, 238 (45%) reported cost of cannabinoid therapy as a challenge. Many participants (269; 50.9%) reported using *somewhat less*” or *much less* owing to the challenges they experienced. Furthermore, participants used varying methods to consume MC, with 740 (74.3%) using a smoking method, 386 (38.8%) using a vaping device, 538 (54%) utilizing store-bought edibles, 175 (17.6%) using concentrates, 173 (17.4%) using homemade food products, 155 (15.6%) using topicals, 138 (13.9%) using tinctures, 112 (11.2%) utilizing cannabis beverages, 13 (1.3%) indicating other, and 0% using suppositories. There was no significant variation in methods to consume MC between states with different legal status.

There were 544 (54.6%) participants who reported that their medical provider recommended MC. A proportion of participants, 79 (18.2%) of those who responded to the question, reported challenges in obtaining a medical card, whereas 34 (43%) participants cited finding a provider to recommend MC as a challenge. There were 566 (56.8%) participants reporting that they disclosed MC use to their primary care provider (PCP), whereas 183 (32.3% of those who responded to the question) of those participants disclosed their cannabis use because the PCP asked them directly. Participants who did not share the cannabis use with their PCP did not do so owing to fear of the provider not approving of their use (94; 39.8% of those who responded), state legality (64; 27.1% of those who responded), and other reasons. The reason participants stated for not sharing their cannabis use with their PCPs was that *It is illegal in my state*, with 14.7% of participants residing in no legal access states, 38.4% of participants residing in MC permitted states, and 27.4% of participants residing in nonmedical/MC permitted states reporting *It is illegal in my state* as the reason not to share their cannabis use with their PCPs.

Table 2 reports how the different formulations of cannabis were obtained; physical dispensaries were the primary source to obtain MC. Furthermore, illicit means were rarely utilized to obtain MC. Table 3 provides the frequency of using each method, with smoking MC daily being the top choice among participants (442; 59.9%). Using a vape (171; 44.3%) or concentrate (72; 41.1%) was the second and third most commonly used daily formulations among participants, respectively. Growing cannabis and acquisition through illicit means for a vaping device had a significant association with state legality (*p*=0.009 and *p*=0.04, respectively).

**Table 2:** Methods of Obtaining Medical Cannabis

Cannabis formulation	Physical dispensary	Online/delivery	Grow my own	Friend or family member	Illicit means	Other
Smoke	442 (59.9%)	133 (18.0%)	88 (11.9%)	311 (42.1%)	45 (6.1%)	5 (0.7%)
Vape	262 (67.9%)	108 (28.0%)	25 (6.5%)	100 (25.9%)	8 (2.1%)	11 (2.8%)
Concentrate	133 (76.0%)	42 (24.0%)	22 (12.6%)	47 (26.9%)	5 (2.9%)	2 (1.1%)
Edibles	377 (70.1%)	138 (25.7%)	27 (05.0%)	149 (27.7%)	8 (1.5%)	19 (3.5%)
Homemade Food Products	92 (53.2%)	40 (23.1%)	32 (18.5%)	72 (41.6%)	3 (1.7%)	4 (2.3%)
Tinctures	99 (72.8%)	37 (27.2%)	16 (11.8%)	25 (18.4%)	1 (0.7%)	1 (0.7%)
Topicals	109 (69.0%)	58 (36.7%)	9 (5.7%)	31 (19.6%)	1 (0.6%)	3 (1.9%)
Beverages	74 (67.3%)	43 (39.1%)	12 (10.9%)	32 (29.1%)	1 (0.9%)	1 (0.9%)
Other	3 (23.1%)	2 (15.4%)	1 (7.7%)	5 (38.5%)	1 (7.7%)	2 (15.4%)

*Note:* The group frequency and proportion (inside the parentheses) were reported. The proportion represents the number of participants that stated *Yes* versus *No* in the row and column grouping.

**Table 3:** Frequency of Medical Cannabis Use

Cannabis formulation	Daily	Less than once a month	Monthly	Unsure	Weekly
Smoke	442 (59.9%)	57 (7.7%)	50 (6.8%)	14 (1.9%)	175 (23.7%)
Vape	171 (44.3%)	48 (12.4%)	46 (11.9%)	11 (2.8%)	110 (28.5%)
Concentrate	72 (41.1%)	20 (11.4%)	26 (14.9%)	10 (5.7%)	47 (26.9%)
Edibles	132 (24.5%)	138 (25.7%)	113 (21.0%)	17 (3.2%)	138 (25.7%)
Homemade food products	29 (16.8%)	54 (31.2%)	30 (17.3%)	11 (6.4%)	49 (28.3%)
Tinctures	37 (27.2%)	37 (27.2%)	31 (22.8%)	5 (3.7%)	26 (19.1%)
Topicals	53 (33.5%)	29 (18.4%)	32 (20.3%)	8 (5.1%)	36 (22.8%)
Beverages	21 (19.1%)	36 (32.7%)	13 (11.8%)	6 (5.5%)	34 (30.9%)
Other	1 (7.7%)	6 (46.2%)	2 (15.4%)	3 (23.1%)	1 (7.7%)

*Note:* The group frequency and proportion (inside the parentheses) were reported. The proportion represents the number of participants that stated Yes versus No in the row and column grouping.

Respondents reported MC use for anxiety (656; 65.9%), depression (478; 48%), insomnia (374; 37.6%), chronic pain (370; 37%), migraines (248; 24.9%), post-traumatic stress disorder (237; 23.8%), bipolar disorder (178; 17.9%), attention-deficit hyperactivity disorder (171; 17.2%), and acute pain (155; 15.6%) as the most common ailments (multiple selections were permitted)—all other reported ailments were less than 10% of respondents. Bipolar disorder, degenerative neurologic conditions, and acute pain showed a significant association with state legality (*p*=0.008, *p*=0.04, and *p*=0.01, respectively). In no legal access states, 24% of participants used MC for bipolar disorder compared with 18% of participants in MC permitted states and 15% of participants in nonmedical/MC permitted states. For degenerative neurologic condition, 2.2% of participants in no legal access states used MC compared with 6.1% of participants in MC permitted states and 6.1% of participants nonmedical/MC permitted states. For acute pain, 19% of participants used MC in nonmedical/MC permitted states compared with 15% of participants in no legal access states and 11% of participants in MC permitted states.

Participants reported their perceived efficacy of MC on their primary ailment on a 5-point Likert scale (*a great deal, quite a bit, a moderate amount, slightly*, or *not at all*). There were 322 (32.3%) participants who claimed that MC was a great deal effective; quite a bit was selected by 352 (35.3%) participants. There were 223 (22.4%) participants who indicated that it helped to a moderate amount. Seventy-seven (7.7%) participants selected slightly, and 22 (2.2%) participants said cannabis did not at all help with their primary ailment. Perceived efficacy by MC users did not significantly vary between states of different legal status.

Table 4 demonstrates the observed side effects of MC for each method of use. Although side effects varied depending on the method of consumption, the most commonly reported side effects were increased appetite, dry mouth, and none. Results show that side effects vary significantly on the basis of method of consumption: no side effects (chi-square=216, *p*<0.001), dry mouth (Fisher’s *p*<0.001), paranoia (Fisher’s *p*=0.032), unwanted drowsiness (Fisher’s *p*=0.002), increased appetite (Fisher’s *p*<0.001), and other side effects (Fisher’s *p*=0.044). The use of tinctures (54%), topicals (81.67%), and beverages (60%) had the highest rates of no side effects, whereas smoking cannabis had the highest rate (74.3%) of side effects. In addition, anxiety, dizziness, and cannabinoid hyperemesis syndrome had no difference in rate by method of consumption. Results showed that no side effects due to smoking and state legality were related to each other (*p*=0.022). In states with no legal access, 30.3% of participants reported having no side effects due to smoking cannabis, compared with 27.4% of participants in states with nonmedical/MC permitted and 19% of participants in states with MC permitted.

**Table 4:** Side Effects Related to Medical Cannabis Use

*n* (%)	None	Anxiety	Dry mouth	Paranoia	Dizziness	Unwanted drowsiness	Increased appetite	Cannabinoid hyperemesis syndrome	Other
Smoke	190 (25.7%)	78 (10.6%)	372 (50.4%)	71 (9.6%)	60 (8.1%)	98 (13.3%)	351 (47.6%)	10 (1.4%)	16 (2.2%)
Vape	144 (37.3%)	43 (11.1%)	135 (35.0%)	25 (6.5%)	30 (7.8%)	37 (9.6%)	124 (32.1%)	3 (0.8%)	15 (3.9%)
Concentrate	61 (34.9%)	18 (10.3%)	64 (36.6%)	13 (7.4%)	16 (9.1%)	26 (14.9%)	64 (36.6%)	4 (2.3%)	8 (4.6%)
Edibles	244 (45.4%)	61 (11.3%)	112 (20.8%)	44 (8.2%)	47 (8.7%)	79 (14.7%)	147 (27.3%)	5 (0.9%)	13 (2.4%)
Homemade food products	76 (43.9%)	20 (11.6%)	41 (23.7%)	17 (9.8%)	15 (8.7%)	34 (19.7%)	56 (32.4%)	2 (1.2%)	4 (2.3%)
Tinctures	74 (54.4%)	12 (8.8%)	28 (20.6%)	11 (8.1%)	10 (7.4%)	20 (14.7%)	31 (22.8%)	1 (0.7%)	2 (1.5%)
Topicals	129 (81.6%)	8 (5.1%)	13 (8.2%)	5 (3.2%)	5 (3.2%)	9 (5.7%)	15 (9.5%)	1 (0.6%)	1 (0.6%)
Beverages	66 (60.0%)	20 (18.2%)	15 (13.6%)	3 (2.7%)	13 (11.8%)	9 (8.2%)	21 (19.1%)	2 (1.8%)	1 (0.9%)
Other	6 (46.2%)	1 (7.7%)	2 (15.4%)	2 (15.4%)	0 (0.0%)	1 (7.7%)	2 (15.4%)	0 (0.0%)	2 (15.4%)

*Note:* The group frequency (*n*) and proportion (inside the parentheses as a percent) were reported. The proportion represents the number of participants that stated *Yes* versus *No* in the row and column grouping.

## DISCUSSION

These data provide evidence that legislation against cannabis use does curtail the use of MC. Although many aspects of MC use did not vary between states of different legal status, there were important differences in the MC experience, which had ramifications for patients on MC. Of reported challenges and barriers, fear of legal recourse and social judgment stand out. Many of those who did not inform their PCP withheld said facts out of fear of disapproval and legal concerns. Most respondents reported having challenges utilizing MC. Cost of cannabis was shared as a challenge for many patients, with half who reported it as a challenge stating that they used less cannabis owing to it.

Respondents were asked through a variety of questions to provide an overview of their health, in general and in relation to MC. Respondents’ health data ranged drastically, representing the diverse population utilizing cannabis. In relation to their primary ailment, of the 996 respondents, 545 (54.7%) reported MC being effective for their ailment, supporting the overall efficacy of MC for a variety of conditions. There were no significant differences in side-effect frequency or severity between those in legal and illegal states. However, there were significant differences between side effects from different means of consuming cannabis. Smoking had by far the most frequent occurrence of side effects than other means such as tinctures. This supports conventional clinical practices that favor tinctures over potentially hazardous smoking or vaping.^20^^,^^21^ Despite this, inhalants/smoking, vaping, and concentrates were the top 3 ways of utilizing MC. For certain side effects such as cannabinoid hyperemesis syndrome, there were no significant variations in frequency by form of consumption.

Although overall state legality was not a significant factor in most variables regarding MC use, there were some notable exceptions. For example, there were some significant findings regarding what conditions MC was used for in states with legal access to MC versus states without legal access. Patients on MC who resided in states without legal access reported using cannabis for bipolar disorder more often, with an almost 10% increase compared with that in nonmedical/medical usage–permitting states. This may be due to the legal concerns mentioned earlier discouraging patients on MC from disclosing their use to their PCP, who may inform them of the possible negative interactions between cannabis and mood disorders.^22^ Inversely, individuals in nonmedically or medically permissive states reported more than twice as high a rate of using MC for degenerative neurologic conditions, for which MC has promising potential.^23^ These 2 instances highlight the importance of cannabis education for both patients and medical care providers—education on the uses, benefits, and risks of MC has been shown to improve attitudes toward it, and generally, patients report better outcomes when using MC with the guidance of a healthcare provider.^24^^,^^25^ A commonly cited reason for not disclosing MC use to one’s PCP was related to the perceived legal status of cannabis in their state, irrespective of its actual legality in their reported state. Patients on MC in MC permitted or nonmedical/MC permitted states were more likely to cite state illegality as a barrier to disclosing their MC use to their PCP, as opposed to individuals in no legal access states. This may indicate the prevalence of misconceptions among patients on MC about the laws in their states and perhaps about what is regarded as MC. These results underscore how legal concerns can influence individuals to withhold medical information from their PCP. This information is particularly concerning considering that cannabis could potentially interact negatively with other medications being used and possibly exacerbate symptoms of the treated ailment. Conversely, patients with conditions that may warrant MC who do not currently use it could experience significant benefits from its treatment. The data collected in this study support the efficacy of MC but also highlights the points at which patients on MC face challenges in undertaking their cannabinoid therapy.

### Limitations

As with any project employing a self-report survey for data collection, ensuring the accuracy of the responses is a critical priority. This survey incorporated multiple attention check questions to address this issue, ensuring that all completed surveys could be regarded as having been carefully considered rather than completed hastily. Similarly, respondents may have feared reporting activities that are illegal in their state, leading to dishonest responses. Respondents may also not have an accurate understanding of their own cannabis use habits, leading to underreporting or overreporting. Although the responses were slightly skewed to female, there was significant diversity in the sample in regard to race, gender, and age. Further research should be concerned with analyzing specifics of the data, such as how efficacy varies between those undertaking cannabinoid therapy with and without clinician guidance, including regression analyses to evaluate causal relationships.

## CONCLUSIONS

This research sheds light on the use of MC across states with varying legal access, emphasizing its therapeutic potential alongside the barriers patients face. Despite differing legal frameworks, the data reveal widespread MC usage, with significant links between state legality and the conditions treated. These findings highlight the critical need for clear communication between patients and healthcare providers, particularly concerning potential side effects and interactions with other treatments. Moreover, the challenges of cost and access in restrictive states highlight the urgent need for policy reforms to enhance patient care. Further research is vital to deepen an understanding of the clinical implications of MC use and the influence of legislation on access and health outcomes.

## Appendix Supplementary materials

Image, application 1
