# Supplementary material for: Medical Cannabis Utilization Across States With Varying Legal Status
Source: AJPM Focus. 2025 Jul 22;4(5):100396. doi: 10.1016/j.focus.2025.100396 (PMC12448028; doi:10.1016/j.focus.2025.100396)
Supplement: Supplementary file 1 [file mmc1.docx]

**Appendix: Medical Cannabis Survey (accompanying Medical Cannabis Utilization Across States with Varying Legal Status, Deanna Romero et al., AJPM Focus, 2025(**

Please read the following information before you decide whether or not to participate.

1. The purpose of this research study is to understand cannabis use and the impact of cannabis policies throughout the United States.
2. You will be asked about cannabis use and your opinions about cannabis use. You will be asked some questions about other health-related behaviors and your background. Parents will be asked about their children’s cannabis use.
3. This survey will take about 20 minutes to complete.
4. You must be at least 18 years of age to participate in this study.
5. Participation is voluntary. If eligible to complete the survey after preliminary questions, your offered incentive will be provided if you complete it. You can choose to end the study at any point; however, you will not receive compensation if you do not complete the survey. All questions must be answered in order to receive compensation.
6. At the end of the survey, depending on your responses to the questions, you may be asked to participate in an interview, to be scheduled at a later date. This is also completely voluntary, and your completion of this survey and your offered incentive are independent of your agreeing to be interviewed. Depending on the legal status of cannabis in your state, you may be disclosing illegal actions accessing and using cannabis if you choose to provide your name and contact information.
7. Findings may be shared with other researchers and/or published. However, all provided information will be grouped with responses from other participants. This will ensure that there will be no way to identify participants individually.
8. The findings may help policymakers better understand the specific impacts of medical cannabis laws.
9. It is possible you may experience some emotional discomfort while answering some of the questions in this survey. You may discontinue answering questions at any time if your discomfort becomes intolerable. The same is true for those who volunteer and are selected for the interviews.
10. The data will be stored for a minimum of 7 years on a secure server at California State University Channel Islands.
11. The collected data will be anonymous and confidential. Researchers will not collect or use internet protocol (IP) addresses or other information which could link your responses to your computer or electronic device. However, when information is transmitted over the internet, privacy cannot be guaranteed. There is always a risk your responses may be intercepted by a third party (e.g., government agencies, hackers).
12. This study has been reviewed and received ethics clearance through the Institutional Review Board at California State University Channel Islands.
13. By providing your consent, you are not waiving your legal rights or releasing the investigator(s) or involved institution(s) from their legal and professional responsibilities.
14. For all other questions about the study, please contact Dr. Thomas A. Clobes at [thomas.clobes@csuci.edu](mailto:thomas.clobes@csuci.edu) or 805-437-3308.

Your initiation of this survey serves as your consent to participate in the study.

**Demographics (D) (Q18)**

The first set of questions are to learn about who you are, where you live, and similar information. All of the responses will be kept confidential and are anonymous.

| D3 | What is your age? [Drop down menu] |
| --- | --- |
| D4 | How do you best describe yourself? [Select all that apply if multiracial]   1. American Indian/Alaska Native 2. Asian 3. Black/African American 4. Native Hawaiian or other Pacific Islander 5. White   Other (fill in blank) |
| D5 | Are you of Hispanic/Latino origin?   1. Yes 2. No   Unsure |
| D1 | What gender do you identify as?   1. Male 2. Female 3. Non-binary/third gender 4. Other: [fill in the blank] |
| D13 | What is your marital status?   1. Single/never married 2. Married 3. Domestic Partnership/civil union 4. Separated 5. Divorced 6. Widowed |
| D15 | How many children do you have? [Drop down menu] |
| D14 | What is your sexual identity/sexual orientation?   1. Heterosexual 2. Homosexual 3. Bisexual 4. Other [Fill in blank] |
| D9 | What is your citizenship status?   1. U.S. citizen by birth 2. U.S. citizen by naturalization 3. Documented permanent resident 4. Documented temporary resident 5. Undocumented resident |
| D9.1 | [Do not display to those who selected “U.S. citizen by birth” in D9]  What country were you born in? [free text entry] |
| D10 | What state do you live in? [prepopulated response dropdown menu] |
| D7 | What is your current living situation? I live with: [Select all that apply]:   1. Parent(s)/guardian(s) 2. Roommate(s) 3. Partner/Spouse 4. Children 17 years and under 5. Children 18 year and older 6. Live alone 7. Other [fill in the blank] |
| D8 | Which of the following best describes where you live? [Select all that apply]   1. Detached single-family home 2. Attached house (townhome, semi-detached house) 3. Multiple unit building (apartment, condo) 4. Share accommodations (rooming house, residence/dorm/retirement home) 5. Unhoused/homeless/no permanent residence 6. Other [fill in the blank] |
| D11 | What is the highest degree or level of education you have completed?   1. Did not attend high school 2. Some high school 3. High school 4. Some college 5. Trade school/vocational degree 6. Associates degree 7. Bachelor’s degree 8. Master’s degree 9. Doctoral degree |
| D12 | What is the **total** annual income (before taxes) for **all individuals** living in your household?   1. Less than $25,000 per year 2. $25,001 - $50,000 per year 3. $50,001 - 75,000 per year 4. $75,001 - $100,000 per year 5. $100,001 - $150,000 per year 6. $150,001 - $200,000 per year 7. $200,001 or more per year |
| D16 | What is your employment status? [Check all that apply]   1. Employed full-time 2. Employed part-time 3. Not employed, looking for work 4. Not employed, not looking for work 5. Retired 6. Disabled, temporarily 7. Disabled, permanently |
| D17 | Are you a student?   1. Yes, a full-time student 2. Yes, a part-time student 3. No |
| D18 | What is your primary work industry (or what was it, if no longer working)?   1. Accountant, banking, or finance 2. Agriculture (other than cannabis) 3. Cannabis industry 4. Construction 5. Creative arts/design 6. Data processing 7. Education 8. Engineering 9. Entertainment 10. Food services 11. Finance 12. Firefighter 13. Health care 14. Hotel services 15. Information services/computing/software 16. Law enforcement or security 17. Legal Services 18. Leisure, sport, or tourism 19. Marketing, advertising, or public relations 20. Media 21. Military 22. Non-profit charity 23. Public service 24. Publishing 25. Religious 26. Student (with no other employment) 27. Transportation 28. Utilities 29. Other (enter response) |
| D19 | Have you ever served in active duty in the U.S. Armed Forces, military reserves, or National Guard?   1. Yes 2. No |
| D20 | What is your political party affiliation?   1. Democratic Party 2. Republican Party 3. Libertarian Party 4. Independent Party/no party affiliation 5. Not registered to vote 6. Other [enter response] |
| D21 | How would you describe your political views?   1. Very liberal 2. Somewhat Liberal 3. Moderate 4. Somewhat Conservative 5. Very Conservative |
| D22 | If applicable, what is your religion?   1. Protestant 2. Roman Catholic 3. Mormon/LDS 4. Orthodox 5. Jewish 6. Muslim 7. Buddhist 8. Hindu 9. No religion 10. Other [enter response] |

**Cannabis Use (CU) (Q12)**

The following questions will ask about your personal use of cannabis. If you are a parent, there will also be questions about your children’s cannabis use; please remember, all of this information is confidential. We will use the term cannabis (also known as marijuana, pot, weed, hash, kush, and other names) to refer to all of the different forms of the plant and its preparations, including, but not limited to:

- Dried herb
- Edibles
- Oils (e.g., vaped or taken orally)
- Hash or kief
- Concentrates (dab, wax, shatter, budder, etc.)
- Cannabis drinks (tea, cola, etc.)
- Tinctures, lotions, or other cannabis products

| CU1 | For the purposes of this survey, **“medical cannabis”** is defined as cannabis used to treat or aid with some sort of medical issue, whether or not with a recommendation or supervision from a medical provider. For example, using cannabis occasionally to help sleep **would** be considered medical cannabis use.  Do you **currently** use cannabis?   1. Yes, for recreational use only 2. Yes, for medical use only 3. Yes, for both recreational and medical use 4. No |
| --- | --- |
| CU1.2 | [Display to those who said “no” in CU1]  Have you **ever** – even once - used cannabis?   1. Yes, for recreational use only 2. Yes, for medical use only 3. Yes, for both recreational and medical use 4. No |
| Screener Results | [End survey for those who selected “recreational only” OR “no” to CU1 **AND** “no” OR “recreational only” to CU1.2.] |
| CU1.3 | [Display to those who selected “medical” or “both” in CU 1 or CU1.2]  Did you use cannabis for **medical purposes** **prior to the age of 18**?   1. Yes 2. No 3. Unsure |
| CU1.4 | [Display for those who answer “no” to CU1 and “yes” CU1.2]  For what reasons did you stop using cannabis? [Check all that apply]   1. Could not tolerate side effects 2. Friends and family did not approve 3. Primary care provider urged you to quit 4. Cannabis was making health issues worse 5. Employment required regular drug testing 6. Fear of legal, academic, or employment consequences 7. Was never a regular user 8. Felt as if you had no control over the amount you used 9. Moved to a state without legal access 10. Became pregnant or started breastfeeding 11. Was unable to obtain cannabis in a safe and consistent manner 12. Other [Fill in the blank] |
| CU1.5 | [Display for those who answer “no” to CU1 AND CU1.2]  For what reasons have you refrained from using cannabis? [Check all that apply]   1. No interest 2. Fear of legal consequences 3. Concern over reactions from friends and family 4. Limited access to cannabis 5. Use would put employment at risk 6. Concerns regarding interactions with medications 7. Concerns about side effects 8. Financial limitations/cost 9. Other [Fill in the blank] |
| CU2 | [Display to those who entered more than 0 to D15]  Do you have a **child currently *under* the age of 18** who you know uses (or previously used) cannabis?   1. Yes, for recreational use only 2. Yes, for medical use only 3. Yes, for both recreational and medical use 4. No 5. I do not know 6. Do not wish to disclose |
| CU3 | [Display to those who entered more than 0 to D15]  Do you have a **child currently *over* the age of 18** who used cannabis **prior to turning 18**?   1. Yes, for recreational use only 2. Yes, for medical use only 3. Yes, for both recreational and medical use 4. No 5. I do not know 6. Do not wish to disclose |
| CU4 | [Display to those who selected “yes” option to CU1 or CU1.2]  How has cannabis use affected your **physical** health?   1. Significant negative impact 2. Some negative impact 3. No impact 4. Some positive impact 5. Significant positive impact |
| CU5 | [Display to those who selected “yes” to using either cannabis to CU1 and CU1.2]  How has cannabis use affected your **mental** health?   1. Significant negative impact 2. Some negative impact 3. No impact 4. Some positive impact 5. Significant positive impact |

**Current Health Status (CHS) (Q28)**

The next set of questions will ask you about your current physical and mental health.

| Attention Check | This is a question not related to the research project which was intentionally included. Who was the first President of the United State of America?   1. Margaret Thames 2. Neal Tyree 3. Tom Jones 4. Peter Ford 5. George Washington |
| --- | --- |
| CHS1 | How would you rate your current health status?   1. Very poor 2. Poor 3. Fair 4. Good 5. Very good |
| CHS1.1 | How would you rate your overall quality of life?   1. Very poor 2. Poor 3. Fair 4. Good 5. Very good |
| CHS2 | Do you have any of the following conditions? [Select all that apply]   1. Autism spectrum disorder 2. Autoimmune Diseases 3. Cachexia/anorexia 4. Cancer pain 5. Chronic pain 6. Degenerative neurological conditions 7. Depression/anxiety/PTSD 8. Insomnia 9. Irritable bowel syndrome (IBS) 10. Migraines 11. Seizures 12. Other [fill in blank] |
| CHS3 | Please select any and all medications you currently use? [Select all that apply]   1. Sedatives/Sleep aides — such as Ambien, Lunesta and Benadryl 2. Anti-anxiety — such as Xanax, Valium and Librium 3. Antidepressants — such as Zoloft, Prozac and Lexapro 4. Pain medications — such as codeine, Percocet and Vicodin 5. Anticonvulsants/Anti-seizure  — such as Tegretol, Topamax and Depakene 6. Anticoagulants Blood thinners — such as Coumadin, Plavix and heparin 7. Antipsychotics - such as Abilify, risperdal, and seroquel 8. NSAIDs — such as Naproxen, Ibuprofen, Celebrex 9. None |
| CHS4 | Has a medical provider ever diagnosed you with a substance use disorder (substance addiction)?   1. Yes 2. No |
| CHS4.1 | [Display to those who selected “yes” in CHS4]  Which substance use disorder?   1. Opioid Use Disorder 2. Cannabis Use Disorder/Marijuana Use Disorder 3. Nicotine Use Disorder 4. Stimulant Use Disorder 5. Sedative Use Disorder 6. Hallucinogen Use Disorder 7. Alcohol Use Disorder 8. Other [Fill in the blank] |
| CHS5 | Do you currently or have you ever used any tobacco product?   1. Yes 2. No |
| CHS5.1 | [Display to those who selected “yes” in CHS5]  Are you currently using any tobacco product?   1. Yes 2. No |
| CHS5.7 | [Display to those who selected “no” in CHS5.1]  How long did you use any tobacco product for?   1. Only a brief period of time 2. Less than one year but more than a brief period of time 3. One to five years 4. Five to ten years 5. More than 10 years |
| CHS5.8 | [Display to those who selected “yes” in CHS5.1]  How long did you use any tobacco product for?   1. Only a brief period of time 2. Less than one year but more than a brief period of time 3. One to five years 4. Five to ten years   More than 10 years |
| CHS5.8 | [Display to those who selected “no” in CHS5.1]  When did you stop using any tobacco product?   1. Recently, within the past few months 2. Within the past year but more than a few months ago 3. One to five years ago 4. Five to ten years ago 5. More than 10 years ago |
| CHS6 | Do you currently drink alcohol?   1. Yes 2. No |
| CHS6.1 | [Display to those who selected “yes” to CHS6]  How often do you drink alcohol?   1. Less than once a month 2. 1-3 times a month 3. 1-2 times a week 4. 3-4 times a week 5. Every day or nearly every day |
| CHS6.2 | [Display to those who selected “female” in D2]  Do you ever consume 4 or more drinks in a single day?   1. Yes 2. No |
| CHS6.3 | Display to those who selected “yes” to CHS6.2]  How often do you consume more than four drinks in a single day?   1. Less than once a month 2. Several times per month 3. Once per week 4. Several times per week 5. Daily |
| CHS6.4 | [Display to those who selected “male” in D2]  Do you ever consume 5 or more drinks on a single occasion?   1. Yes 2. No |
| CHS6.5 | [Display to those who selected “yes” to CHS6.4]  How often do you consume more than five drinks in a single day?   1. Less than once a month 2. Several times per month 3. Once per week 4. Several times per week 5. Daily |
| CHS7 | Do you **currently** use any substances other than cannabis, tobacco, and alcohol (mushrooms/psilocybin, illegally obtained prescription medications, MDMA/ecstasy, heroin, LSD, etc.)?   1. Yes 2. No |
| CHS7.1 | [Display to those who selected “yes” to CHS7]  Which substances do you use? [Free text] |
| CHS7.2 | [Display to those who selected “no” to CHS7]  Have you **previously, but not currently**, used any substances other than cannabis, tobacco, and alcohol (mushrooms/psilocybin, illegally obtained prescription medications, methamphetamine, MDMA/ecstasy, heroin, LSD, etc.)?   1. Yes 2. No 3. Prefer not to answer |
| CHS7.3 | [Display to those who selected “yes” to CHS7.2]  What substances did you use? [Free text] |
| CHS8 | How would you rate your diet?   1. Very poor 2. Poor 3. Fair 4. Good 5. Very good |
| CHS9 | How often do you exercise?   1. Never 2. Rarely 3. Once a week 4. 1-2 times a week 5. 3 or more times a week |
| CHS10 | How often do you have an appointment for yourself with your primary care provider?   1. Never but I do have a primary care provider 2. Never and I do not have a primary care provider 3. Rarely 4. Once a year 5. 1-2 times a year 6. more than three times a year |

**Current Medical Marijuana Use (CMMU)** [Only display to those who selected “medical” or “both” in CU1]

You indicated that you currently use cannabis for **medical purposes**. The following questions will be specific to your **current** **medical** cannabis use. **(Q81)**

| CMMU1 | Do you **currently** have a state-issued **medical** **cannabis card**?   1. Yes 2. No 3. Used to have a card, but have not maintained it |
| --- | --- |
| CMMU1.1 | [Display to those who selected “no” to CMMU1]  Have you **ever** had a state-issued **medical** **cannabis card**?   1. Yes 2. No |
| CMMU1.2 | [Display to those who selected “yes” to CMMU1 in CMMU1.1]  Did you experience any challenges or difficulties obtaining your **medical** **cannabis card**?   1. Yes 2. No |
| CMMU24 | [Display to those who selected “yes” to CMMU1.2]  What challenges did you experience obtaining your **medical** **cannabis card**? [Select all that apply]   - 1. Difficulty finding a provider to recommend medical marijuana   2. Cost of seeing a provider to recommend marijuana too high   3. The cost of the card is too high   4. It took a long time to get the card after applying   5. There is too much paperwork to get the card   6. Other (fill in the blank) |
| CMMU1.3 | [Display to those who selected “no” to CMMU1 AND CMMU1.1]  Why have you chosen to **not** obtain a state-issued **medical** cannabis card? [Select all that apply]   1. I do not know of a provider who would make the official recommendation 2. My request for a medical cannabis recommendation was denied by the healthcare provider 3. Too expensive 4. Not worth the hassle 5. I can easily obtain cannabis from an illicit source (such as, from a friend or family member) 6. I can easily obtain cannabis through a dispensary as a recreational product 7. My state does not have a medical cannabis card program 8. Other [Fill in the blank] |
| CMMU1.4 | [Display to those who selected “yes” to CMMU1 or CMMU1.1]  Why did you choose to obtain a state-issued **medical** **cannabis card**? [Select all that apply]   1. It is required in my state 2. There is no other way for me to obtain cannabis 3. Pay less taxes on the cannabis products I acquire 4. To enroll in a compassionate care program and receive free cannabis 5. Prefer to use official channels to obtain my cannabis 6. Able to acquire more product having it 7. Able to acquire product with higher concentration of THC having it 8. Other |
| CMMU1.5 | [Display to those who selected “no” to CMMU1 AND “yes” to CMMU1.1]  Why did you decide to not renew your **medical** **cannabis card**? [select all that apply]   1. Too expensive 2. Not worth the hassle 3. I can easily obtain cannabis without it 4. Other [Fill in the blank] |
| CMMU2 | Regardless of having a medical card or not, was **medical** cannabis recommended to you by a licensed medical provider?   1. Yes 2. No |
| CMMU2.1 | [Display to those who selected “yes” to CMMU2]  In what modality did you see the medical provider who recommended **medical** cannabis to you? [Select all that apply]   1. In-person 2. Using telehealth technology (internet video) 3. Over the phone 4. Never met with the provider, only answered online questionnaire 5. Other [fill in the blank] |
| CMMU2.2 | [Display to those who selected “yes” to CMMU2]  How often did you see the provider who recommended your **medical** cannabis use?   1. Weekly 2. Monthly 3. Quarterly 4. Once a year 5. Only saw them once 6. Other [fill in the blank] |
| CMMU3 | Does your primary care provider, if not the provider who recommended cannabis, know about your **medical** cannabis use?   1. Yes 2. No 3. My primary care provider is who recommended medical cannabis 4. I do not have a primary care provider 5. I have a primary care provider, but have not seen them since starting medical cannabis |
| CMMU3.1 | [Display to those who selected “yes” to CMMU3]  Why did you choose to share this information with your primary care provider? [Select all that apply]   1. They asked me directly 2. It was a question on a medical information survey they had me complete 3. I was concerned about interactions with my other medications 4. I felt they needed to know 5. Other (fill in the blank) |
| CMMU3.2 | [Display to those who selected “no” to CMMU3]  What reason(s) do you have for not sharing this information with your provider? [Select all that apply]   1. Fear provider would not approve 2. Provider recommended against medical cannabis 3. It is illegal in my state and I am discrete about my use 4. A family member attends my doctor’s appointment with me and I do not what them to know about my use 5. Other (fill in the blank) |
| CMMU4 | How old were you when you first tried **medical** cannabis (in years)? [Drop down menu] |
| CMMU5 | What was your motivation to try **medical** cannabis for the first time? [Select all that apply]   1. Curiosity 2. Recommendation of a provider 3. Recommendation of a healthcare professional other than a provider 4. As an alternative to a conventional pharmaceutical treatment 5. Recommendation of a friend or family member 6. Conducted your own research and discovered it may be helpful 7. Thought it was worth experimenting how it affects medical conditions 8. Other [Fill in the blank] |
| CMMU7.1 | How often do you use **medical** cannabis of any type?   1. Multiple times a day 2. Daily 3. Weekly 4. Monthly 5. Less than once a month |
| CMMU7.2 | What is the total amount of time you have used **medical** cannabis (in years and months)? [Restrict answers] |
| CMMU8 | To what extent have you ever experienced challenges obtaining **medical** cannabis?   1. Not at all 2. Slightly 3. A moderate amount 4. Quite a bit 5. A great deal |
| CMMU8.1 | [Display to those who selected “slightly” or more in CMMU8]  What challenges have you experienced obtaining cannabis for **medical** purposes? [Select all that apply]   1. Could not find a source to obtain cannabis from 2. Difficulty finding a trustworthy source 3. It is illegal in my state 4. Could not afford it 5. Dispensaries not near my home 6. Did not know what product(s) to use 7. Other: |
| CMMU8.2 | [Display to those who selected “slightly” or higher to CMMU8]  How did these challenges obtaining **medical** cannabis impact the amount you use?   1. Use much less 2. Use somewhat less 3. Use about the same 4. Use somewhat more 5. Use much more |
| CMMU9 | What ailments do you use **medical** cannabis for? (Select all that apply)   1. ADD/ADHD 2. Alcohol or other drug use 3. Anxiety 4. Autism spectrum disorder 5. Autoimmune diseases 6. Bipolar disorder/Mania/Borderline personality disorder 7. Cancer pain 8. Cachexia 9. Degenerative neurological conditions 10. Depression 11. Eating disorder 12. Insomnia 13. Irritable bowel syndrome (IBS) 14. Migraines 15. Pain (acute; short durations lasting less than six months) 16. Pain (chronic; lasting more than six months) 17. Post-traumatic stress disorder (PTSD) 18. Psychosis/Dissociative identity disorder 19. Schizophrenia 20. Seizures 21. Other (enter response - must enter response) |
| CMMU9.11 | [Piped text from CMMU9]  Of the ailments you identified treating with **medical** cannabis, what would you consider your **primary** ailment you use medical cannabis for?   1. [Include ailments from CMMU9.11] 2. I consider more than one ailment equally primary |
| CMMU9.12 | [Piped text from CMMU9; for this who selected “I consider more than one ailment equally primary”]  Which of the ailments you identified treating with **medical** cannabis, do you consider your **primary** ailments you use medical cannabis for?   1. [Include ailments from CMMU9.11]   [For those who select multiple ailments, display the “least fill” ailment for the appropriate questions below] |
| CMMU9.1 ***Move to before CMMU9.11 | [Display to those who selected “pain” in CMMU9]  Have you **ever** used cannabis instead of opioids or other prescription medication to manage your pain?   1. Yes 2. No 3. Unsure |
| CMMU9.2 | [Display to those who selected “yes” in CMMU9.1]  **In the past 12 months,** have you used cannabis instead of opioids or other prescription medication to manage your pain?   1. Yes 2. No 3. Unsure |
| CMMU9.3 | [Piped text from CMMU9; repeat for each ailment]  When considering [**specific ailment**], how well does cannabis relieve your symptoms?   1. Not at all 2. Slightly 3. A moderate amount 4. Quite a bit 5. A great deal |
| CMMU9.4 | [Piped text from CMMU9; if selected more than one ailment] You indicated you use **medical** cannabis for [**identified ailments**].  Do you use the same cannabis product regardless of the ailment you are using it for?   1. Yes 2. No |
| The following questions will ask you about the specific methods you use to consume medical cannabis. The questions are similar to one another but are asking about different periods of time. Please pay close attention to exactly what the question is asking. | |
| CMMU9.7 | [Piped text from CMMU9]  When thinking specifically about [**specific ailment**], what method(s) of consumption for **medical use** of cannabis have you used? [Select all that apply]   1. Smoke 2. Vape 3. Concentrate 4. Edibles (store bought) 5. Homemade food products 6. Tinctures (drops under the tongue) 7. Topicals (lotions, oils, etc.) 8. Beverages 9. Other (enter response) |
| CMMU9.9 | [Display to those who selected “smoke” in CMMU9.8]  What method(s) do you **currently** use for **smoking** **medical** cannabis? [Select all that apply]   1. Bong 2. Pre-rolled joints 3. Joints you roll yourself 4. Pipe 5. Blunts 6. Other (enter response) |
| CMMU10 | [Piped text from CMMU9.8; display for each route of administration]  What is your preferred method(s) of **acquiring** **[route of administration]** cannabis for **medical use**? [Select all that apply]   1. Physical dispensary location 2. Online/Delivery 3. Grow my own 4. Friend or family member 5. Illicit means (such as, from a friend or family member) [please provide details] 6. Other [enter response] |
| CMMU11 | [Piped text from CMMU9.8; display for each route of administration]  In what location(s) do you use [**specific type**] **medical** cannabis? [Select all that apply]   1. Inside a home you own 2. Inside a home you are renting 3. Inside at a friend or family member’s home 4. Outside at a home you own 5. Outside at a home you rent 6. Outside at a friend or family member’s home 7. Public spaces 8. In a car 9. Other [Fill in the blank] |
| Attention Check | This is a question not related to the research project that was intentionally included. For this question, please select “Neutral.”   1. Strongly disagree 2. Somewhat disagree 3. Neutral 4. Somewhat agree 5. Strongly agree |
| CMMU12 | [Piped text from CMMU9.6; display for each route of administration]  When thinking about when you [**route of administration**] , what side effects, if any, have you experienced while using cannabis for **medical** purposes? [Select all that apply]   1. None 2. Anxiety 3. Dry mouth 4. Paranoia 5. Dizziness 6. Unwanted drowsiness 7. Increased appetite 8. Cannabinoid hyperemesis syndrome (repeated, severe bouts of vomiting) 9. Other [Fill in the blank] |
| CMMU13 | [Piped text from CMMU12; display for each side effect],  When thinking about [**side effect**] experienced while using [**route of administration**], how has that side effect impacted the amount of **medical** cannabis you use?   1. Use much less 2. Use a little less 3. The amount has not changed 4. Use a little more 5. Use much more |
| CMMU13.1 | Have you ever been hospitalized or needed to seek care at an emergency department or urgent care because of your cannabis use?   1. Yes 2. No 3. Unsure |
| CMMU13.2 | [Display to those who selected “yes” in CMMU13.1]  Please provide details on the medical care you sought (symptoms, type of care, location of care, diagnosis, treatment, date, etc.). [Free text] |
| CMMU14 | **In the last 12 months**, how has the total amount of cannabis you use for **medical** purposes on average changed?   1. Use much less 2. Use a little less 3. The amount has not changed 4. Use a little more 5. Use much more |
| CMMU15 | [Piped text from CMMU9]  When thinking about [**ailment**], what formulation of **medical** cannabis do you prefer?   1. THC only 2. CBD only 3. THC/CBD 4. Unsure 5. Other (fill in blank) |
| CMMU16 | Do you know the specific amount of THC & CBD that is in the **medical** cannabis product you use?   1. Yes 2. No |
| CMMU16.1 | [Piped text from CMMU9.8; display for each type of administration; Display to those who selected “yes” to CMMU16; If selected “smoke,” “vape,” or “concentrate”]  What **potency** of **THC** is in the [**route of administration**] you are generally consuming for **medical** purposes?   1. No THC 2. Some THC but below 10% THC 3. 11-20% THC 4. 21-35% THC 5. 36-70% THC 6. 71% or higher THC |
| CMMU16.2 | [Piped text from CMMU9.8; display for each type of administration; Display to those who selected “yes” to CMMU16; If selected “smoke,” “vape,” or “concentrate”]  What **potency** of **CBD** is in the [**route of administration**] you are generally consuming for medical purposes?   1. No CBD 2. Some CBD but below 10% CBD 3. 11-20% CBD 4. 21-35% CBD 5. 36-70% CBD 6. 71% or higher CBD |
| CMMU17 | [Display to those who selected “pre-rolled joints” or “rolls joint themselves” in CMMU9.9]  Please choose the joint size closest to what you typically smoke for **medical** purposes:  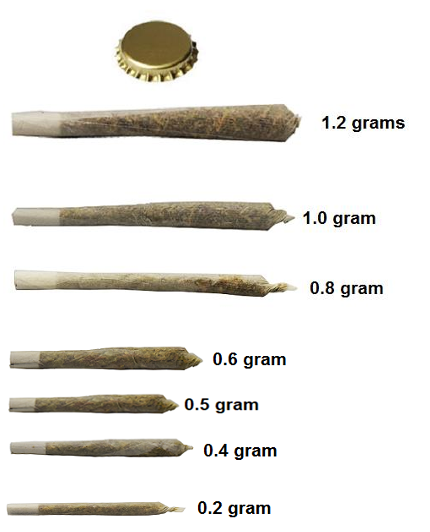   - More than 1.2 grams - Unsure |
| CMMU17.1 | [Display to those who selected “bong,” “blunt,” or “pipe” in CMMU9.9]  Please choose the quantity of cannabis closest to what you typically smoke in one sitting for **medical** purposes:  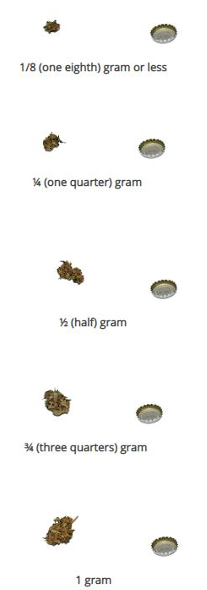  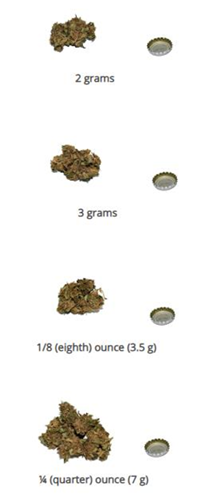   - More than ¼ (quarter) ounce (7 grams) - Unsure |
| CMMU17.2 | [Piped text from CMMU9.8; ask for each type of administration]  When thinking specifically about **medical use**, how often do you use [**route of administration**] cannabis?   1. Less than once a month 2. Monthly 3. Weekly 4. Daily 5. Unsure |
| CMMU17.3 | [Piped text from CMMU9.8; Display to those who selected “smoke,” “vape,” or “concentrate” in CMMU9.8 AND “daily” in CMMU17.2]  **On average**, how many times **per da**y do you use [**route of administration**] cannabis for **medical use**? [Free text] |
| CMMU17.4 | [Piped text from CMMU9.8; Display to those who selected “smoke,” “vape,” or “concentrate” in CMMU9.8 AND “weekly” in CMMU17.2]  **On average**, how many days **per week** do you use [**route of administration**] cannabis for **medical use**? [Free text] |
| CMMU17.5 | [Piped text from CMMU9.8; If “smoke,” “vape,” or “concentrate” in CMMU9.8 AND “monththly” in CMMU17.2]  **On average**, how many **days per month** do you use [**route of administration**] cannabis for **medical use**? [Free text] |
| CMMU17.6 | [Piped text from CMMU9.8; If “smoke,” “vape,” or “concentrate” in CMMU.8 AND “less than once per month” in CMMU17.2]  **Approximately**, how many **days over the past 12 months** did you use [**route of administration**] cannabis for **medical use**? [Free text] |
| CMMU17.7 | [Piped text from CMMU9.9; If “bong,” “pre-rolled joints,” “rolled joint themselves,” “pipe,” or “blunts" in CMMU9.9]  How much of the [**route of administration**] cannabis do you typically smoke in one sitting for **medical use**?   1. One-fourth of it 2. Half of it 3. Three-fourths of it 4. All of it 5. Unsure |
| CMMU17.8 | [Display to those who selected “vape” in CMMU9.8]  Vape pens and cartridges typically come in two sizes: 0.5 grams and 1.0 grams. What size do you typically acquire?   1. 0.5 grams 2. 1.0 grams 3. Unsure |
| CMMU17.9 | [Display to those who selected “vape” in CMMU9.8 AND in “weekly” or “daily” CMMU17.2]  For **medical purposes**, how many vape cartridges or disposable vape pens do you use in an **average month**?   1. Less than one 2. 1 3. 2 4. 3 5. 4 6. 5 7. 6 8. 7 9. 8 10. 9 11. 10 12. 11 13. 12 14. 13 15. 14 16. 15 17. 16 18. 17 19. 18 20. 19 21. 20 22. More than 20 23. Unsure |
| CMMU17.10 | [Display to those who selected “vape” in CMMU9.8 AND “less than one a month” or “one or two times a month” CMMU17.2]  For **medical purposes**, how many vape cartridges or disposable vape pens do you use in an **average year**?   1. Less than one 2. 1 3. 2 4. 3 5. 4 6. 5 7. 6 8. 7 9. 8 10. 9 11. 10 12. 11 13. 12 14. 13 15. 14 16. 15 17. 16 18. 17 19. 18 20. 19 21. 20 22. More than 20 23. Unsure |
| CMMU17.11 | [Piped text from CMMU9.7; Display for each type of administration; Display to those who selected “yes” to CMMU16; If selected “edibles,” “homemade,” “tinctures,” beverages,” “suppositories,” or “topicals” in CMMU9.7]  For **medical use,** what amount of **THC** is in the [**route of administration**] cannabis you typically use **each time**?   1. No THC 2. Less than 5 mg THC 3. 6 to 10 mg THC 4. 11 to 20 mg THC 5. 21 to 50 mg THC 6. 51 to 100 mg THC 7. Over 100 mg THC 8. Unsure |
| CMMU17.12 | [Piped text from CMMU9.7; Display for each type of administration; Display to those who selected “yes” to CMMU16; If selected “edibles,” “homemade,” “tinctures,” beverages,” “suppositories,” or “topicals” in CMMU9.7]  For **medical use,** what amount of **CBD** is in the [**route of administration**] you typically use **each time**?   1. No CBD 2. Less than 5 mg CBD 3. 6 to 10 mg CBD 4. 11 to 20 mg CBD 5. 21 to 50 mg CBD 6. 51 to 100 mg CBD 7. Over 100 mg CBD 8. Unsure |
| CMMU17.13 | [Piped text from in CMMU9.7; If “edibles,” “homemade,” “tinctures,” beverages,” “suppositories,” or “topicals” in in CMMU9.7 AND “daily” in CMMU17.2]  **On averag**e, how many times **per da**y do you use [**route of administration**] cannabis for **medical use**? [Answers restricted] |
| CMMU17.14 | [Piped text from in CMMU9.7; If “edibles,” “homemade,” “tinctures,” beverages,” “suppositories,” or “topicals” in in CMMU9.7 AND “weekly” in CMMU17.2]  **On average**, how many **days per week** do you use [**route of administration**] cannabis for **medical use**? [Free text] |
| CMMU17.15 | [Piped text from in CMMU9.7; If “edibles,” “homemade,” “tinctures,” beverages,” “suppositories,” or “topicals” in in CMMU9.7 AND “monthly” in CMMU17.2]  **On average**, how many **days per month** do you use [**route of administration**] cannabis for **medical use**? [Free text] |
| CMMU17.16 | [Piped text from in CMMU9.7; If “edibles,” “homemade,” “tinctures,” beverages,” “suppositories,” or “topicals” in in CMMU9.7 AND “less than once a month” in CMMU17.2]  **Approximately**, how many **days over the past 12 months** did you use [**route of administration**] cannabis for **medical use**? [Free text] |
| CMMU18 | Are you familiar with lesser known components of **medical** cannabis such as CBN, CBG, terpenes, or flavonoids?   1. Not at all familiar 2. Somewhat familiar 3. Moderately familiar 4. Very familiar 5. Extremely familiar |
| CMMU18.1 | [Display to those who selected anything other than “not at all familiar” to CMMU18]  Do you acquire **medical** cannabis products with specific components in mind other than THC or CBD?   1. Yes 2. No |
| CMMU18.2 | [Piped text from CMMU9; Display to those who selected anything other than “not at all familiar” to CMMU18]  When thinking of [**specific ailment**], what component(s) of **medical** cannabis, other than THC and CBD, do you desire in the products you acquire?   1. CBN 2. CBG 3. Specific terpenes [fill in the blank] 4. Specific flavonoids [fill in the blank] 5. Other [fill in the blank] 6. Unsure |
| CMMU19 | Have you ever sought advice from cannabis dispensary staff (i.e. budtenders) when acquiring **medical** cannabis, whether the acquisition is made at the dispensary or elsewhere?   1. Never 2. Rarely 3. Sometimes 4. Often 5. All the time |
| CMMU19.1 | [Display to those who selected anything other than “never” CMMU19.1]  Regarding **medical** cannabis, how helpful was the advice you have received from cannabis dispensary staff?   1. Not at all helpful 2. Slightly helpful 3. Moderately helpful 4. Very helpful 5. Extremely helpful |
| CMMU19.2 | [Display to those who selected anything other than “never” CMMU19.1]  How closely have your **medical** cannabis product acquisitions matched the recommendations from the cannabis dispensary staff?   1. Not at all 2. Slightly 3. A moderate amount 4. Quite a bit 5. A great deal |
| CMMU20 | How much do you spend per month, on average, on the **medical** cannabis you consume?   1. Less than $50 2. $50 - 100 3. $100 - $200 4. $200 - $400 5. Over $400 |
| CMMU21 | To what extent, if any, do you think your **medical** cannabis use is an issue or problematic?   1. Not at all 2. Slightly 3. A moderate amount 4. Quite a bit 5. A great deal |
| CMMU21.1 | To what extent, if any, have friends or family members communicated that your **medical** cannabis use is an issue or problematic?   1. Not at all 2. Slightly 3. A moderate amount 4. Quite a bit 5. A great deal |
| CMMU21.2 | [Display to those who selected anything other than “not at all” to CMMU21.1]  What feedback have friends or family members shared with you about your **medical** cannabis use? [Select all that apply]   1. Interfering with work or education 2. Interfering with romantic relationship 3. Interfering with personal, non-romantic relationships 4. Spending too much money on cannabis 5. Not able to be fully mentally present when needed 6. Causing other health issues 7. Irrational fear of cannabis 8. Lack of education on cannabis 9. Concerns about it being a “gateway drug” 10. Other [Fill in the blank] |
| CMMU21.3 | [Display to those who selected anything other than “not at all” to CMMU21.1]  How has feedback communicated to you by your friends or family impacted the amount of **medical cannabis** you use?   1. Use much less 2. Use somewhat less 3. Use about the same 4. Use somewhat more 5. Use much more |
| CMMU21.4 | Regardless of feedback from some friends or family, to what extent have other friends and family supported your use of **medical cannabis**?   1. Not at all 2. Slightly 3. A moderate amount 4. Quite a bit 5. A great deal |
| CMMU21.5 | [Display to those who selected anything other than “not at all” to CMMU21.4]  How has support communicated to you by your friends or family impacted the amount of **medical cannabis** you use?   1. Use much less 2. Use somewhat less 3. Use about the same 4. Use somewhat more 5. Use much more |
| CMMU21.6 | [Display to those who selected anything other than “not at all” to CMMU21.4]  In what ways have friends or family members been supportive of your medical cannabis use? [Select all that apply]   1. Purchased cannabis products for me 2. Helped you with dosing or administration of your cannabis product 3. Provided emotional support 4. Provided transportation to health care professional providing a medical cannabis recommendation 5. Provided transportation to cannabis dispensary 6. Assisted you in completing the paperwork for a medical card 7. Defended your use of medical cannabis to other friends and/or family 8. Other [Fill in the bank] |
| CMMU22 | Have you ever tried to quit using **medical** cannabis?   1. Yes, I successfully quit 2. Yes, but I was not successful 3. No, but I have thought about it 4. No, I have never considered quitting |
| CMMU22.1 | [Display to those who selected either “yes” in CMMU22]  Why did you try to quit using **medical** cannabis? [Select all that apply]   1. Could not tolerate side effects 2. Friends and family did not approve 3. Primary care provider urged you to quit 4. Cannabis was making health issues worse 5. Employment required regular drug testing 6. Felt as if you had no control over the amount you used 7. Moved to a state without legal access 8. Became pregnant or started breastfeeding 9. Other [Fill in the blank] |
| CMMU22.2 | [Display to those who selected either “yes” in CMMU22]  How difficult was it for you to try to quit using **medical** cannabis?   1. Not at all 2. Slightly 3. A moderate amount 4. Quite a bit 5. A great deal |
| CMMU23 | Is there anything else about your use of **medical** cannabis that you would like to share with the researchers? [Free response] |
| CMMU25 | [Display to those who selected “no” to CMMU1 AND “yes” to CMMU1.1]  Why have you chosen to **not** renew your state-issued **medical** cannabis card? [Select all that apply]   1. My provider who made the official recommendation was no longer available 2. My request for a medical cannabis recommendation was denied by the healthcare provider 3. Too expensive 4. Not worth the hassle 5. I can easily obtain cannabis from an illicit source (such as, from a friend or family member) 6. I can easily obtain cannabis through a dispensary as a recreational product 7. I moved to another state and it does not have a medical cannabis card program 8. Other [Fill in the blank] |

**Past Medical Cannabis Use (PMMU)** [Only display to those who selected “medical” or “both” in CU1.2]

You indicated that you previously used cannabis for **medical purposes**. The following questions will be specific to your **past** use of **medical** cannabis. **(Q 74)**

| PMMU1 | Do you **currently** have a state-issued **medical** **cannabis card**?   1. Yes 2. No 3. Used to have a card, but have not maintained it |
| --- | --- |
| PMMU1.1 | [Display to those who selected “no” to PMMU1]  Have you **ever** had a state-issued **medical** **cannabis card**?   1. Yes 2. No 3. Used to have a card, but have not maintained it |
| PMMU1.2 | [Display to those who selected “yes” to PMMU1 in PMMU1.1]  Did you experience any challenges or difficulties obtaining your **medical** **cannabis card**?   1. Yes 2. No |
| PMMU23 | [Display to those who selected “yes” to PMMU1.2]  What challenges did you experience obtaining your **medical** **cannabis card**? [Select all that apply]   - 1. Difficulty finding a provider to recommend medical marijuana   2. Cost of seeing a provider to recommend marijuana too high   3. The cost of the card is too high   4. It took a long time to get the card after applying   5. There is too much paperwork to get the card   6. Other (fill in the blank) |
| PMMU1.3 | [Display to those who selected “no” to PMMU1 AND PMMU1.1]  Why did you choose to **not** obtain a state-issued **medical** cannabis card? [Select all that apply]   1. I do not know of a provider who would make the official recommendation 2. My request for a medical cannabis recommendation was denied by the healthcare provider 3. Too expensive 4. Not worth the hassle 5. I can easily obtain cannabis from an illicit source (such as, from a friend or family member) 6. I can easily obtain cannabis through a dispensary as a recreational product 7. My state does not have a medical cannabis card program 8. Other [Fill in the blank] |
| PMMU1.4 | [Display to those who selected “yes” to PMMU1 or PMMU1.1]  Why did you choose to obtain a state-issued **medical** **cannabis card**? [Select all that apply]   1. It is required in my state 2. There is no other way for me to obtain cannabis 3. Pay less taxes on the cannabis products I acquire 4. To enroll in a compassionate care program and receive free cannabis 5. Prefer to use official channels to obtain my cannabis 6. Able to acquire more product having it 7. Able to acquire product with higher concentration of THC having it 8. Other |
| PMMU2 | Regardless of having a medical card or not, was **medical** cannabis recommended to you by a licensed medical provider?   1. Yes 2. No |
| PMMU2.1 | [Display to those who selected “yes” to PMMU2]  In what modality did you see the medical provider who recommended **medical** cannabis to you? [Select all that apply]   1. In-person 2. Using telehealth technology (internet video) 3. Over the phone 4. Never met with the provider, only answered online questionnaire 5. Other [fill in the blank] |
| PMMU2.2 | [Display to those who selected “yes” to PMMU2]  How often did you see the provider who recommended your **medical** cannabis use?   1. Weekly 2. Monthly 3. Quarterly 4. Once a year 5. Only saw them once 6. Other [fill in the blank] |
| PMMU3 | Did your primary care provider, if not the provider who recommended cannabis, know about your **medical** cannabis use?   1. Yes 2. No 3. My primary care provider is who recommended medical cannabis 4. I do not have a primary care provider 5. I have a primary care provider, but have not seen them since starting medical cannabis |
| PMMU3.1 | [Display to those who selected “yes” to PMMU3]  Why did you choose to share this information with your primary care provider? [Select all that apply]   1. They asked me directly 2. It was a question on a medical information survey they had me complete 3. I was concerned about interactions with my other medications 4. I felt they needed to know 5. Other (fill in the blank) |
| PMMU3.2 | [Display to those who selected “no” to PMMU3]  What reason(s) did you have for not sharing this information with your provider? [Select all that apply]   1. Fear provider would not approve 2. Provider recommended against medical cannabis 3. It is illegal in my state and I am discrete about my use 4. A family member attends my doctor’s appointment with me and I do not what them to know about my use 5. Afraid I might lose my health insurance coverage 6. Concerned that my provider might stop treating me or stop prescribing other medications 7. Other (fill in the blank) |
| PMMU4 | How old were you when you first tried **medical** cannabis (in years)? [Drop down menu] |
| PMMU5 | What was your motivation to try **medical** cannabis for the first time? [Select all that apply]   1. Curiosity 2. Recommendation of a provider 3. Recommendation of a healthcare professional other than a provider 4. As an alternative to a conventional pharmaceutical treatment 5. Recommendation of a friend or family member 6. Conducted your own research and discovered it may be helpful 7. Thought it was worth experimenting how it affects medical conditions 8. Other [Fill in the blank] |
| PMMU7 | When was the last time you used **medical** cannabis of any type? [Restrict entry to date format]   1. Enter date |
| PMMU7.1 | How often did you use **medical** cannabis of any type?   1. Multiple times a day 2. Daily 3. Weekly 4. Monthly 5. Less than once a month |
| PMMU7.2 | What is the total amount of time you have used **medical** cannabis (in years and months)? [Restrict answers] |
| PMMU8 | To what extent have you ever experienced challenges obtaining **medical** cannabis?   1. Not at all 2. Slightly 3. A moderate amount 4. Quite a bit 5. A great deal |
| PMMU8.1 | [Display to those who selected “slightly” or more in PMMU8]  What challenges did you experience obtaining cannabis for **medical** purposes? [Select all that apply]   1. Could not find a source to obtain cannabis from 2. Difficulty finding a trustworthy source 3. It is illegal in my state 4. Could not afford it 5. Dispensaries not near my home 6. Did not know what product(s) to use 7. Other: |
| PMMU8.2 | [Display to those who selected “slightly” or higher to CMMU8]  How did these challenges obtaining **medical** cannabis impact the amount you use?   1. Use much less 2. Use somewhat less 3. Use about the same 4. Use somewhat more 5. Use much more |
| PMMU9 | What ailments did you use **medical** cannabis for? (Select all that apply)   1. ADD/ADHD 2. Alcohol or other drug use 3. Anxiety 4. Autism spectrum disorder 5. Autoimmune diseases 6. Bipolar disorder/Mania/Borderline personality disorder 7. Cancer pain 8. Cachexia 9. Degenerative neurological conditions 10. Depression 11. Eating disorder 12. Insomnia 13. Irritable bowel syndrome (IBS) 14. Migraines 15. Pain (acute; short durations lasting less than six months) 16. Pain (chronic; lasting more than six months) 17. Post-traumatic stress disorder (PTSD) 18. Psychosis/Dissociative identity disorder 19. Schizophrenia 20. SeizuresOther (enter response - must enter response) |
| PMMU9.1 | [Display to those who selected “pain” in PMMU9]  Have you **ever** used cannabis instead of opioids or other prescription medication to manage your pain?   1. Yes 2. No 3. Unsure |
| PMMU9.2 | [Piped text from PMMU9; repeat for each ailment]  When considering [**specific ailment**], how well did cannabis relieve your symptoms? [Repeat for each selected ailment]   1. Not at all 2. Slightly 3. A moderate amount 4. Quite a bit 5. A great deal |
| PMMU9.3 | [Piped text from CMMU9; if selected more than one ailment]  You indicated you used **medical** cannabis for [**identified ailments**]. Did you use the same cannabis product regardless of the ailment you are using it for?   1. Yes 2. No |
| The following questions will ask you about the specific methods you used to consume medical cannabis. The questions are similar to one another but are asking about different periods of time. Please pay close attention to exactly what the question is asking. | |
| PMMU9.5 | [Piped text from PMMU9]  When thinking specifically about [**specific ailment**], what method(s) of consumption of cannabis did you use?   1. Smoke 2. Vape 3. Concentrate 4. Edibles (store bought) 5. Homemade food products 6. Tinctures (drops under the tongue) 7. Topicals (lotions, oils, etc.) 8. Beverages 9. Suppositories 10. Other (enter response) |
| PMMU9.6 | [Display to those who selected “smoke” in PMMU9]  What method(s) did use for **smoking** **medical** cannabis? [Select all that apply]   1. Bong 2. Pre-rolled joints 3. Joints you roll yourself 4. Pipe 5. Blunts 6. Other (enter response) |
| PMMU9.7 | [Piped text from PMMU9.4; display for each route of administration]  What was your preferred method(s) of **acquiring** **[route of administration**] cannabis for **medical use**? [Select all that apply]   1. Physical dispensary location 2. Online/Delivery 3. Grow my own 4. Friend or family member 5. Illicit means (such as, from a friend or family member) [please provide details] 6. Other [enter response] |
| PMMU10 | [Piped text from PMMU9.4; display for each route of administration]  In what location(s) did you use [**specific type**] **medical** cannabis? [Select all that apply]   1. Inside a home you own 2. Inside a home you are renting 3. Inside at a friend or family member’s home 4. Outside at a home you own 5. Outside at a home you rent 6. Outside at a friend or family member’s home 7. Public spaces 8. In a car 9. Other [Fill in the blank] |
| PMMU11 | [Piped text from PMMU9.4; display for each route of administration]  When thinking about when you [**route of administration**] cannabis, what side effects, if any, did you experience while using cannabis for **medical** purposes? [Select all that apply]   1. None 2. Anxiety 3. Dry mouth 4. Paranoia 5. Dizziness 6. Unwanted drowsiness 7. Increased appetite 8. Cannabinoid hyperemesis syndrome (repeated, severe bouts of vomiting) 9. Other [Fill in the blank] |
| PMMU11.1 | [Piped text from 9MMU11; display for each route of administration]  When thinking about [**side effect**] experienced while using [**route of administration**], how did that side effect impact the amount of **medical** cannabis you use? [Repeat for each side effect]   1. Use much less 2. Use a little less 3. The amount has not changed 4. Use a little more 5. Use much more |
| PMMU12 | Have you ever been hospitalized or needed to seek care at an emergency department or urgent care because of your cannabis use?   1. Yes 2. No 3. Unsure |
| PMMU12.1 | [Display to those who selected “yes” in PMMU12]  Please provide details on the medical care you sought (symptoms, type of care, location of care, diagnosis, treatment, date, etc.). [Free text] |
| PMMU13 | [Piped text from PMMU9]  When thinking about [**ailment**], what formulation of **medical** cannabis did you prefer?   1. THC only 2. CBD only 3. THC/CBD 4. Unsure 5. Other (fill in blank) |
| PMMU14 | Do you know the specific amount of THC & CBD that is in the **medical** cannabis product you used?   1. Yes 2. No |
| PMMU14.1 | [Piped text from PMMU9.4; display for each type of administration; Display to those who selected “yes” to PMMU14; If selected “smoke,” “vape,” or “concentrate”]  What **potency** of **THC** was in the [**route of administration**] you are generally consuming for **medical** purposes?   1. No THC 2. Some THC but below 10% THC 3. 11-20% THC 4. 21-35% THC 5. 36-70% THC 6. 71% or higher THC |
| PMMU14.2 | [Piped text from PMMU9.4; display for each type of administration; Display to those who selected “yes” to PMMU14; If selected “smoke,” “vape,” or “concentrate”]  What **potency** of **CBD** was in the [**route of administration**] you are generally consuming for medical purposes?   1. No CBD 2. Some CBD but below 10% CBD 3. 11-20% CBD 4. 21-35% CBD 5. 36-70% CBD 6. 71% or higher CBD |
| PMMU14.3 | [Display to those who selected “pre-rolled joints” or “rolls joints themselves” in PMMU9.6]  Please choose the joint size closest to what you typically smoked for **medical** purposes:  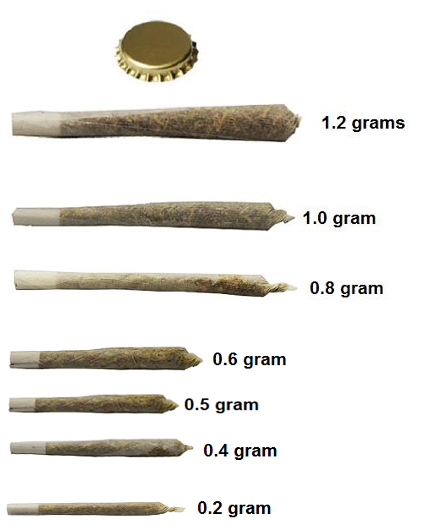   - More than 1.2 grams - Unsure |
| PMMU14.4 | [Display to those who selected “bong,” “blunt,” or “pipe” in PMMU9.6]  Please choose the quantity of cannabis closest to what you typically smoked in one sitting for **medical** purposes:  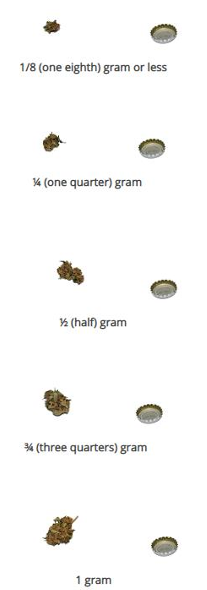  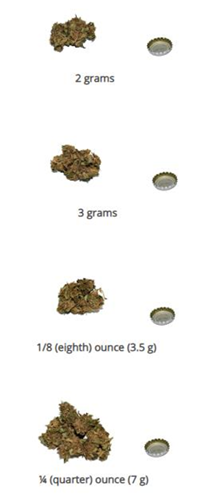   - More than ¼ (quarter) ounce (7 grams) - Unsure |
| PMMU14.5 | [Piped text from PMMU9.5; ask for each type of administration]  When thinking specifically about **medical use**, how often did you use **[route of administration**] cannabis?   1. Less than once a month 2. Monthly 3. Weekly 4. Daily 5. Unsure |
| PMMU14.6 | [Piped text from PMMU9.5; Display to those who selected “smoke,” “vape,” or “concentrate” in PMMU9.5 AND “daily” in PMMU14.5]  **On average**, how many times **per day** did you use [**route of administration**] cannabis for **medical use**? [Free text] |
| PMMU14.7 | [Piped text from PMMU9.5; Display to those who selected “smoke,” “vape,” or “concentrate” in PMMU9.5 AND “weekly” in PMMU14.5]  **On average**, how many days **per week** did you use [**route of administration**] cannabis for **medical use**? [Free text] |
| PMMU14.8 | [Piped text from PMMU9.5; Display to those who selected “smoke,” “vape,” or “concentrate” in PMMU9.5 AND “monthly” in PMMU14.5]  **On average**, how many **days per month** did you use [**route of administration**] cannabis for **medical use**? [Free text] |
| PMMU14.9 | [Piped text from PMMU9.6; If “bong,” “pre-rolled joints,” “rolled joint themselves,” “pipe,” or “blunts" in PMMU9.6]  How much of the [**route of administration**] did you typically smoke in one sitting for **medical use**?   1. One-fourth of it 2. Half of it 3. Three-fourths of it 4. All of it 5. Unsure |
| Attention Check | This is a question not related to the research project that was intentionally included: for this question, please select “Neutral.”   1. Strongly disagree 2. Somewhat disagree 3. Neutral 4. Somewhat agree 5. Strongly agree |
| PMMU14.10 | [Display to those who selected “vape” in PMMU9.5]  Vape pens and cartridges typically come in two sizes: 0.5 grams and 1.0 grams. What size did you typically acquire?   1. 0.5 grams 2. 1.0 grams 3. Unsure |
| PMMU14.11 | [Display to those who selected “vape” in PMMU9.5 AND in “weekly” or “daily” PMMU14.5]  For **medical purposes,** how many vape cartridges or disposable vape pens did you use in an **average month**?   1. Less than one 2. 1 3. 2 4. 3 5. 4 6. 5 7. 6 8. 7 9. 8 10. 9 11. 10 12. 11 13. 12 14. 13 15. 14 16. 15 17. 16 18. 17 19. 18 20. 19 21. 20 22. More than 20 23. Unsure |
| PMMU14.12 | [Display to those who selected “vape” in PMMU9.5 AND “less than one a month” or “one or two times a month” PMMU14.5]  For **medical purposes,** how many vape cartridges or disposable vape pens did you use in an **average year**?   1. Less than one 2. 1 3. 2 4. 3 5. 4 6. 5 7. 6 8. 7 9. 8 10. 9 11. 10 12. 11 13. 12 14. 13 15. 14 16. 15 17. 16 18. 17 19. 18 20. 19 21. 20 22. More than 20 23. Unsure |
| PMMU14.13 | [Piped text from PMMU9.5; Display for each type of administration; Display to those who selected “yes” to PMMU14; If selected “edibles,” “homemade,” “tinctures,” beverages,” “suppositories,” “suppositories,” or “topicals” in PMMU9.5]  For **medical use,** what amount of **THC** is in the [**route of administration**] cannabis you typically use **each time**?   1. No THC 2. Less than 5 mg THC 3. 6 to 10 mg THC 4. 11 to 20 mg THC 5. 21 to 50 mg THC 6. 51 to 100 mg THC 7. Over 100 mg THC 8. Unsure |
| PMMU14.14 | [Piped text from PMMU9.5; Display for each type of administration; Display to those who selected “yes” to PMMU14; If selected “edibles,” “homemade,” “tinctures,” beverages,” “suppositories,” “suppositories,” or “topicals” in PMMU9.5]  For **medical use,** what amount of **CBD** is in the [**route of administration**] you typically used **each time**?   1. No CBD 2. Less than 5 mg CBD 3. 6 to 10 mg CBD 4. 11 to 20 mg CBD 5. 21 to 50 mg CBD 6. 51 to 100 mg CBD 7. Over 100 mg CBD 8. Unsure |
| PMMU14.15 | [Piped text from PMMU9.5; Display for each type of administration; IIf selected “edibles,” “homemade,” “tinctures,” beverages,” “suppositories,” or “topicals” in PMMU9.5 AND “daily” in PMMU14.5]  **On average**, how many times **per day** did you use [**route of administration**] cannabis for **medical use**? [Free text] |
| PMMU14.16 | [Piped text from PMMU9.5; Display for each type of administration; IIf selected “edibles,” “homemade,” “tinctures,” “beverages,” “suppositories,” or “topicals” in PMMU9.5 AND “weekly” in PMMU14.5]  **On average**, how many **days per week** did you use [**route of administration**] cannabis for **medical use**? [Free text] |
| PMMU14.17 | [Piped text from PMMU9.5; Display for each type of administration; IIf selected “edibles,” “homemade,” “tinctures,” beverages,” “suppositories,” or “topicals” in PMMU9.5 AND “monthly” in PMMU14.5]  **On average**, how many **days per month** did you use [**route of administration**] cannabis for **medical use**? [Free text] |
| PMMU14.18 | [[Piped text from PMMU9.5; Display for each type of administration; IIf selected “edibles,” “homemade,” “tinctures,” beverages,” “suppositories,” or “topicals” in PMMU9.5 AND “less than once a month” in PMMU14.5]  **Approximately**, how many **days over a 12-month period** did you use [**route of administration**] cannabis for **medical use**? [Free text] |
| PMMU15 | Are you familiar with lesser known components of **medical** cannabis such as CBN, CBG, terpenes, or flavonoids?   1. Not at all familiar 2. Somewhat familiar 3. Moderately familiar 4. Very familiar 5. Extremely familiar |
| PMMU15.1 | [Display to those who selected anything other than “not at all familiar” to PMMU15]  Did you acquire **medical** cannabis products with specific components in mind other than THC or CBD?   1. Yes 2. No |
| PMMU15.2 | [Piped text from PMMU9; Display to those who selected anything other than “not at all familiar” to PMMU15]  When thinking of [**specific ailment**], what component(s) of **medical** cannabis, other than THC and CBD, did you desire in the products you acquire?   1. CBN 2. CBG 3. Specific terpenes [fill in the blank] 4. Specific flavonoids [fill in the blank] 5. Other [fill in the blank] 6. Unsure |
| PMMU16 | Did you ever seek advice from cannabis dispensary staff (i.e. budtenders) when acquiring **medical** cannabis, whether the acquisition is made at the dispensary or elsewhere?   1. Never 2. Rarely 3. Sometimes 4. Often 5. All the time |
| PMMU16.1 | [Display to those who selected anything other than “never” PMMU16]  Regarding **medical** cannabis, how helpful was the advice you have received from cannabis dispensary staff?   1. Not at all helpful 2. Slightly helpful 3. Moderately helpful 4. Very helpful 5. Extremely helpful |
| PMMU16.2 | [Display to those who selected anything other than “never” PMMU16]  How closely did your **medical** cannabis product acquisitions match the recommendations from the cannabis dispensary staff?   1. Not at all 2. Slightly 3. A moderate amount 4. Quite a bit 5. A great deal |
| PMMU17 | How much did you spend per month, on average, on the **medical** cannabis you consumed?   1. Less than $50 2. $50 - 100 3. $100 - $200 4. $200 - $400 5. Over $400 |
| PMMU18 | To what extent, if any, did you think your **medical** cannabis use was an issue or problematic?   1. Not at all 2. Slightly 3. A moderate amount 4. Quite a bit 5. A great deal |
| PMMU19 | To what extent, if any, have friends or family members communicated that your **medical** cannabis use was an issue or problematic?   1. Not at all 2. Slightly 3. A moderate amount 4. Quite a bit 5. A great deal |
| PMMU19.1 | [Display to those who selected anything other than “not at all” to PMMU19]  What feedback did friends or family members share with you about your **medical** cannabis use? [Select all that apply]   1. Interfering with work or education 2. Interfering with romantic relationship 3. Interfering with personal, non-romantic relationships 4. Spending too much money on cannabis 5. Not able to be fully mentally present when needed 6. Causing other health issues 7. Irrational fear of cannabis 8. Lack of education on cannabis 9. Concerns about it being a “gateway drug” 10. Other [Fill in the blank] |
| PMMU19.2 | [Display to those who selected anything other than “not at all” to PMMU19]  How did feedback communicated to you by your friends or family impacted the amount of **medical cannabis** you use?   1. Use much less 2. Use somewhat less 3. Use about the same 4. Use somewhat more 5. Use much more |
| PMMU20 | Regardless of feedback from some friends or family, to what extent did other friends and family support your use of **medical cannabis**?   1. Not at all 2. Slightly 3. A moderate amount 4. Quite a bit 5. A great deal |
| PMMU20.1 | [Display to those who selected anything other than “not at all” to PMMU20]  How did support communicated to you by your friends or family impact the amount of **medical cannabis** you use?   1. Use much less 2. Use somewhat less 3. Use about the same 4. Use somewhat more 5. Use much more |
| PMMU20.2 | [Display to those who selected anything other than “not at all” to PMMU20]  In what ways were friends or family members been supportive of your medical cannabis use? [Select all that apply]   - - - 1. Purchased cannabis products for me       2. Helped you with dosing or administration of your cannabis product       3. Provided emotional support       4. Provided transportation to health care professional providing a medical cannabis recommendation       5. Provided transportation to cannabis dispensary       6. Assisted you in completing the paperwork for a medical card       7. Defended your use of medical cannabis to other friends and/or family       8. Other [Fill in the bank] |
| PMMU21 | Why did you quit using **medical** cannabis? [Select all that apply]   1. Could not tolerate side effects 2. Friends and family did not approve 3. Primary care provider urged you to quit 4. Cannabis was making health issues worse 5. Employment required regular drug testing 6. Felt as if you had no control over the amount you used 7. Moved to a state without legal access 8. Became pregnant or start breastfeeding 9. Medical issue is no longer a problem 10. Other [Fill in the blank] |
| PMMU21.1 | How difficult was it for you to try to quit using **medical** cannabis?   1. Not at all 2. Slightly 3. A moderate amount 4. Quite a bit 5. A great deal |
| PMMU22 | Is there anything else about your use of **medical** cannabis that you would like to share with the researchers? [Free response] |

**Closing (C) (Q2)**

| C1 | [Only display to those who selected “medical” or “both” in CU1]  The researchers would like to interview medical cannabis patients to discuss the processes used to obtain medical cannabis and reach a better understanding of your experiences. This is an optional activity in addition to completing this survey. If you are randomly selected, the interview will last approximately 60 to 90 minutes, and you will be compensated with a $75 gift certificate for your time. If you agree to be interviewed, your confidentiality and anonymity of your survey responses will be maintained. However, due to the nature of the interview process, your participation and responses in the interview would be confidential but not anonymous. Due to this and depending on the legal status of cannabis in your state, you may be disclosing illegal actions accessing and using cannabis if you choose to provide your name and contact information.   1. Yes, I agree to be interviewed and receive $75 compensation. I understand that my survey information will be kept confidential and anonymous but the interview will not be anonymous. Please click this link to provide your contact information. 2. No, I do not agree to be interviewed.   [For those who choose to participate, their information will be collected separately from the survey data. They will provide their first name, last name, email address, phone number, and state of residence.] |
| --- | --- |
| C2 | Thank you for completing this survey. The researchers appreciate your time and value your input. If you have any questions, you can contact the Principal Investigator at thomas.clobes@csuci.edu |
